# It’s in your hands: How variable perception affects grasping estimates in virtual reality

**DOI:** 10.3758/s13423-021-01916-x

**Published:** 2021-04-05

**Authors:** Megan Rose Readman, Dalton Cooper, Sally A. Linkenauger

**Affiliations:** Department of Psychology, Lancaster University, Lancaster, LA1 4YF UK

**Keywords:** Embodied perception, Grasp ability, Affordance perception, Virtual reality

## Abstract

Successful interaction within one’s environment is contingent upon one’s ability to accurately perceive the extent over which actions can be performed, referred to as action boundaries. As our possibilities for action are subject to variability, it is necessary for individuals to be able to update their perceived action boundaries to accommodate for variance. While research has shown that individuals can update their action boundaries to accommodate for variability, it is unclear how the perceptual system calibrates to this variance to inform our action boundaries. This study investigated the influence of perceptual motor variability by analysing the effect of random and systematic variability on perceived grasp ability in virtual reality. Participants estimated grasp ability following perceptual-motor experience with a constricted, normal, extended, or variable grasp. In Experiment 1, participants experienced all three grasping abilities (constricted, normal, extended) 33% of the time. In Experiment 2 participants experienced the constricted and normal grasps 25% of the time, and the extended grasp 50% of the time. The results indicated that when perceptual-motor feedback is inconsistent, the perceptual system disregards the frequency of perceptual-motor experience with the different action capabilities and considers each action capability experienced as a type, and subsequently calibrates to the average action boundary experienced by type.

**Supplementary Information:**

The online version contains supplementary material available at 10.3758/s13423-021-01916-x.

## Introduction

In ecological terms, successful interaction within the environment is contingent upon one’s ability to accurately perceive the affordances such an environment provides (Gibson, 1979). Affordances are the opportunities for action for a given organism within a particular environmental context (Gibson, 1979; Heras-Escribano & Pinedo-García, 2018). The extent to which an object affords behaviour is determined by the relationship between the specifications of the object and limitations of our bodies (Proffitt & Linkenauger, 2013). For example, the human hand morphology enables grasping, yet constrains the sizes of objects over which grasping can be performed. This maximum extent of one’s action capability is known as an *action boundary* (Fajen, 2005).

Presumably, the development of knowledge concerning ones action boundaries occurs during infancy (Proffitt & Linkenauger, 2013). For example, 5-month-olds perform 100–250 exploratory hand movements every 10 min (Wallace & Whishaw, 2003). Presumably, this exploration allows infants to learn the visual specification of actions that are possible and impossible, enabling them to become finely attuned to their action boundary (Proffitt & Linkenauger, 2013). By adulthood, individuals are highly accurate at perceiving the largest block that affords grasping (Graydon et al., 2012; Linkenauger et al., 2012), the smallest aperture that is passable (Warren & Whang, 1987), and the furthest distance that is reachable (Carello et al., 1989; Linkenauger et al., 2009).

Additionally, individuals can flexibly adjust their affordance estimations to account for alterations in action capabilities (Taylor-Covill & Eves, 2016). For example, the minimum aperture participants attempt to pass their hands through increases accordingly when their hand sizes are enlarged by a prosthesis (Ishak et al., 2008) and the minimum doorways perceived to be passable alters in accordance with changes in girth that occur when participants don a pregnancy pack (Franchak & Adolph, 2014b). While our perceptual system seemingly recalibrates following changes in constraints, previous research has focussed on stable changes that allow us to gain experience with the visual specification of the altered action boundary. However, there are circumstances in which continuous unstable variance in individuals’ abilities occurs. In these cases, an individual’s experience provides them with inconsistent information as to the actions they can and cannot perform. Consider recovery from a broken thumb. While ability to grasp is initially compromised, one’s ability to perform grasping actions will recover in accordance with the rate of healing. Unfortunately, how our perceptual system determines the action boundary following this inconsistency remains unclear.

Successful action can be conceptualised as a binary function, categorised by the ability to succeed or fail in action performance. Accordingly, one might assume that the perception of action capability is also a binary function; whether we perceive an affordance for the action (success) or not (failure) – often measured through terms of an affordance threshold (Franchak et al., 2012). However, as one’s action capabilities across the same task can vary (Fetters, 2010), affordances should not be presumed as categorical. Rather we should measure affordances in terms of a probabilistic function, whereby the likelihood of success is compared to the cost of failure (Franchak & Adolph, 2014a). Although evidence points towards a system of affordances designed to address this variability, how individuals determine their action boundaries after experiencing this variability remains unclear.

**Fig. 1:**
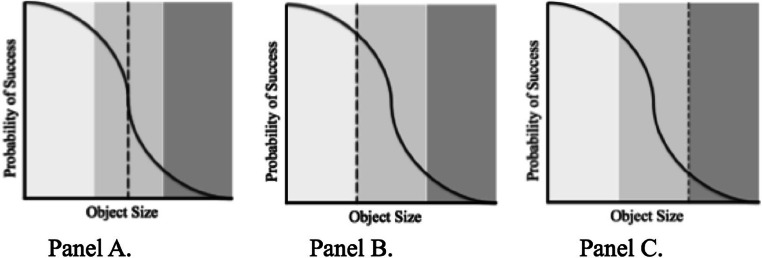
Possible action boundaries that the perceptual system could calibrate to in the face of variability in one’s grasping ability. The dotted perpendicular line in **panel A** represents the action boundary an individual would calibrate to if they were to employ a weighted average approach in which the average of all experience weighted by the degree of occurrence is considered. The dotted perpendicular line in **panel B** represents the action boundary an individual would calibrate to if they were to employ a conservative heuristic in which an individual would calibrate to the most conservative action capability regardless of experience. The dotted perpendicular line in **panel C** represents the action boundary an individual would calibrate to if they were to employ a liberal heuristic in which an individual would calibrate to the most liberal action boundary experienced

It may be that our perceptual system applies a weighted average approach (Loeb & Fishel, 2014) in which the average of all action boundaries experienced weighted by the degree of their occurrence is considered (Körding & Wolpert, 2006). Consider a perceiver who can perform grasps that are 100% of their ability half of the time, and 50% of their ability the remaining time. In assessing the grasp-ability of an object, the perceptual system will calibrate to the average of the perceptual motor feedback, 75% of their maximum ability (see Fig. 1A). While this postulation is in line with the growing application of Bayesian theorem to visual perception (Fiser et al., 2010), the data processing necessary is computationally costly. Therefore, rather than expending vast amounts of resources in calculating the weighted average, the perceptual system may rely on heuristics in this decision-making process (Tversky & Kahneman, 1974).

Two potential heuristic mechanisms are conservative or liberal action boundary placement. When applying a conservative heuristic, individuals may calibrate to the most conservative grasp experienced (see Fig. 1B; Merikle et al., 2001). Because this heuristic would lead to the minimization of failed attempts it would be useful when harmful consequences are associated with failure. Alternatively, individuals may calibrate the most liberal grasp experienced (see Fig. 1C; Buzsaki et al., 2014). Employment of the liberal action boundary would result in most successfully performed actions, but also most failed attempts. Hence, this heuristic would be most useful if a failed attempt had no negative consequence.

Lin et al. (2020) recently analysed the influence of variable perceptual motor experience on action boundary determination for reaching in virtual reality (VR). The authors demonstrated that when perceptual-motor experience for reaching was randomly varied (participants experienced a constricted reach (50% of their maximal ability) 50% of the time and an extended reach (150% of their maximal ability) 50% of the time), perceptions of their action boundary for reaching were biased towards liberal estimations. Notably, this bias also occurred when variability systematically favoured both the constricted reach (participants experienced a constricted reach 50%, normal reach 25%, and extended reach 25% of the time), and the extended reach (participants experienced an extended reach 50%, normal reach 25%, and extended reach 25% of the time).

Whilst Lin et al. (2020) provide insight into the mechanism employed in the face of variability in reaching, reaching is a unsophisticated behaviour that acts to support more intricate actions. Due to this, if failure occurs, an individual can simply re-attempt a reach before completing the more intricate action. Thereby causing reaching to be a low cost-benefit action. Comparatively, grasping is a specialized, complex behaviour (Jeannerod, 1996). In this sense, grasping is a high cost-benefit action as failure may result in breakage or the requirement of the re-performance of several actions. Accounting Franchak and Adolph’s (2014a) view of affordances as probabilistic functions, people will be more likely to have an incautious estimate of their action capabilities for reaching compared to grasping. Therefore, one can question whether the same mechanism would be employed in the face of variability in both reaching and grasping behaviours.

In a series of studies we analysed the influence of both random and systematic variability, favouring a liberal action capability, on individuals’ perceptions of their action boundary for grasping. As it is near impossible to create controlled changes in grasping ability in the real world, perceptual-motor feedback was manipulated in VR. Previous research has shown that participants interact with self-representing, self-animated avatars in virtual environments in a manner comparable to their bodies in the real world (Kilteni et al., 2012; Normand et al., 2011). For example, Funkhouser (2020) observed that individuals overestimate their reaching ability by approximately 15% in VR, which closely corresponds to the 10–20% degree of overestimation observed out of VR (Linkenauger et al., 2009). On these grounds, we expected that participants would interact with the virtual hand in a way comparable to how they would behave in the natural world.

## Experiment 1

In this experiment, we investigated the influence of random variability on the perception of action boundaries for grasping. Participants calibrated to a constricted grasp, a normal grasp, or an extended grasp, or a variable grasp – in which participants experienced all three grasping capabilities 33% of the time, and then provided estimates of their grasp ability for each condition.

## Method

### Participants

G*Power software (Faul et al., 2007) was used to perform an a priori power analysis to ascertain the required sample size to achieve adequate power. The required power (1- β) was set at .80 and the significance level (α) was set to .05. Based on Lin et al. (2020) Experiment 1, where a similar VR paradigm was used to analyse the influence of random variability in reaching ability, we anticipated a large effect size of 0.8. This was deduced as this study obtained a ηp^2^ of .38 with a sample of N =21. For the frequentist parameters defined, a sample size of N = 3 is required to achieve a power of .80 at an alpha of .05.

Thirty Lancaster University students (eight males) aged between 18 and 30 years (*M*_*age*_ *=* 21.00*, SD*_*age*_ = 2.24), participated. All participants received course credit for their participation. All participants were right-handed, had normal or corrected-to-normal vision, and had no known medical history of motoric or rheumatic difficulties.

### Stimuli and apparatus

**Fig. 2:**
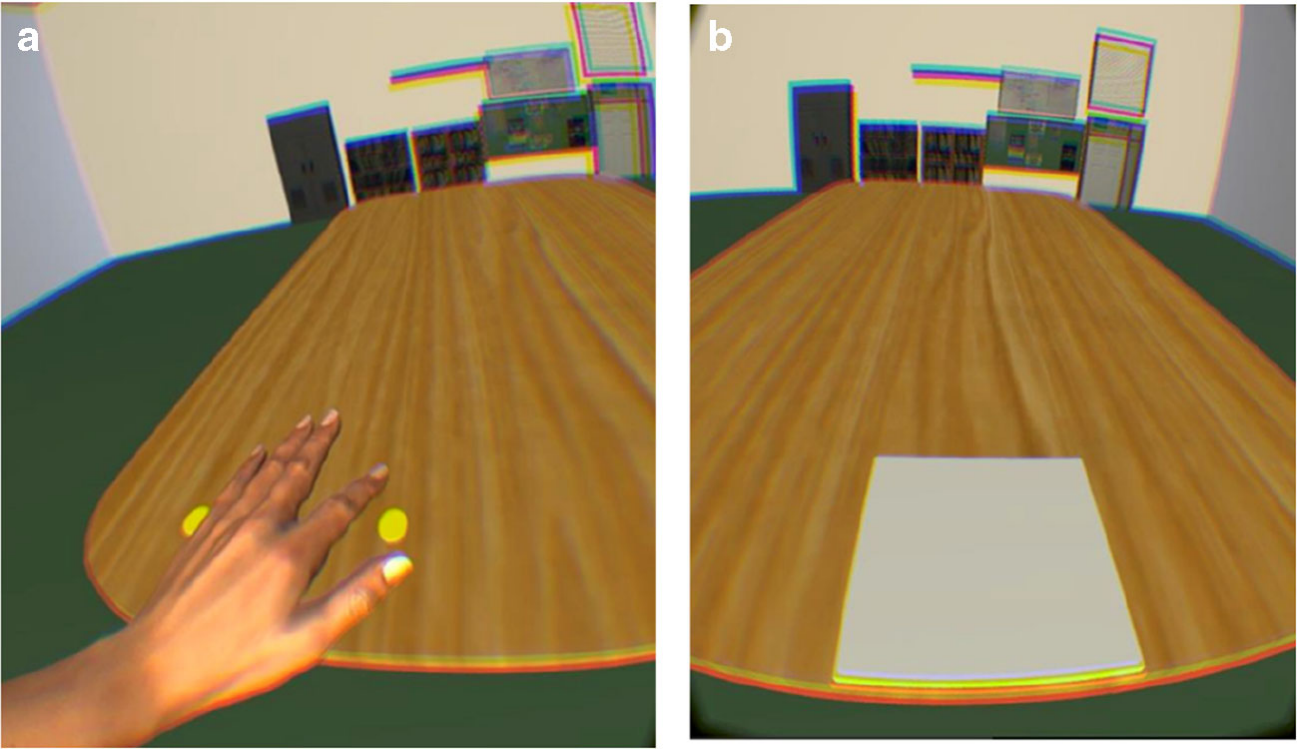
Virtual reality (VR) display presented to participants. **Panel A** depicts the VR set up of the VR display within the calibration phase. **Panel B** depicts the VR set up during the test phase

Participants completed this study sittin at a chair positioned an arm’s length away from the front of a standardised table. A virtual environment was developed in Unity 3D© Gaming Engine with the Leap Motion plugin. The 3D VR colour display comprised a model of a room in which a table was located in the middle (see Fig. 2), and the 3D avatar and camera were placed in front of this table. Upon this table were either two yellow dots (Calibration trials; see Fig. 2A) or a grey block (Test phase trials; see Fig. 2B). The participants viewed the virtual enviornment from a first-person perspective calibrated to their natural eye height. The environment was presented to participants through an Oculus Rift CV1 HMD, which displayed the stereoscopic reality at 2,160 × 1,200 at 90 Hz split over both displays (Binstock, 2015).

The movement of the head was tracked by the head mounted display (HMD) and the perspective of the participant was updated in real time as the participant looked around the environment. The location and position of the participant’s hand was tracked in real time using the Leap Motion hand-tracking sensor mounted on to the Oculus Rift CV1 HMD, and was mapped onto the virtual hand thereby causing the virtual hand to move in correspondence with the natural hand. The avatar hands utilised were taken from the rigged human hand assets provided by Leap Motion for Unity.

### Procedure

Participants were informed that, when estimating graspable objects over the duration of the study, they were to visualise employing a power grasp in which their thumb was placed on one edge of the block and their hand was extended over the surface of the block so that one of their fingers was placed on the parallel edge of the block. Thereafter participants donned the oculus rift HMD with attached Leap Motion Sensor and completed the four experimental conditions (constricted grasp, normal grasp, extended grasp, and variable grasp); the order of completion was counterbalanced across participants.

In the constricted grasp condition, the virtual hand was 50% of the size of their actual hand, therefore constricting the grasp to 50% of the normal grasp ability. In the normal grasp condition, the virtual hand reflected the true size of their actual hand; therefore, grasp ability was 100% of their normal grasp ability. In the extended grasp condition, the virtual hand was 150% of the size of their actual hand thereby extending their grasp ability 50% beyond normal grasp ability. In the variable grasp condition the participants experienced the constricted hand size 33.3% of the time, the normal hand size 33.3% of the time and the extended hand size 33.3% of the time.

Each experiential condition consisted of two phases: the calibration phase and the test phase. The calibration phase consisted of 30 trials in which two parallel dots were presented in front of the participant (see Fig. 2A). Participants were instructed to touch, using their dominant hand, the rightmost dot with their rightmost digit and the leftmost dot with their leftmost digit. Participants were informed that if they could not reach the dot, to position the virtual hand as close to the dots as possible and perform the action as normal. After the participants had performed the action touching both dots, the two dots disappeared and reappeared in a different location on the table. This calibration phase served to provide the participants with the necessary synchronous visual motor information to embody the virtual hand (Kilteni et al., 2012), and provide participants with visual and motor experience regarding the action boundary associated with the virtual hand.

On completion of the calibration phase, participants were instructed to move their hands behind their back out of range of the Leap Motion sensor, which caused the virtual hands to not be visible in the VR. At this time the VR display was altered so that there was a white block on the table (see Fig. 2B). Participants were then instructed to imagine that they were going to grasp the block with one hand from above with a precision grasp. The experimenter then altered the size of the block using the right and left arrow keys of a keyboard until the participant stated the size of the block to reflect the maximum size they believe they would be able to grasp with their dominant hand. Each button press altered the size of the block by 1 cm. Once the participant was satisfied that the size of the block reflected the maximum size they could grasp with their dominant hand, the researcher saved the final size and presented another block. Eight trials were presented; in four of trials the block started at 3 cm and in the remaining four trials the block started at 20 cm. This occurred in order to control for the potential influence previous perception has on later judgements, a phenomenon commonly known as hysteresis (Poltoratski & Tong, 2014).

## Results

One participant was excluded prior to analysis as the results obtained were ±2 SD away from the mean. To analyse the influence of random variability in perceptual motor experience on perceptions of grasping ability, a 4 × 2 repeated-measures ANOVA: 4 (Action capability: Constricted, Normal, Extended, Variable) × 2 (Block Size: Small, Large) was conducted.

**Fig. 3:**
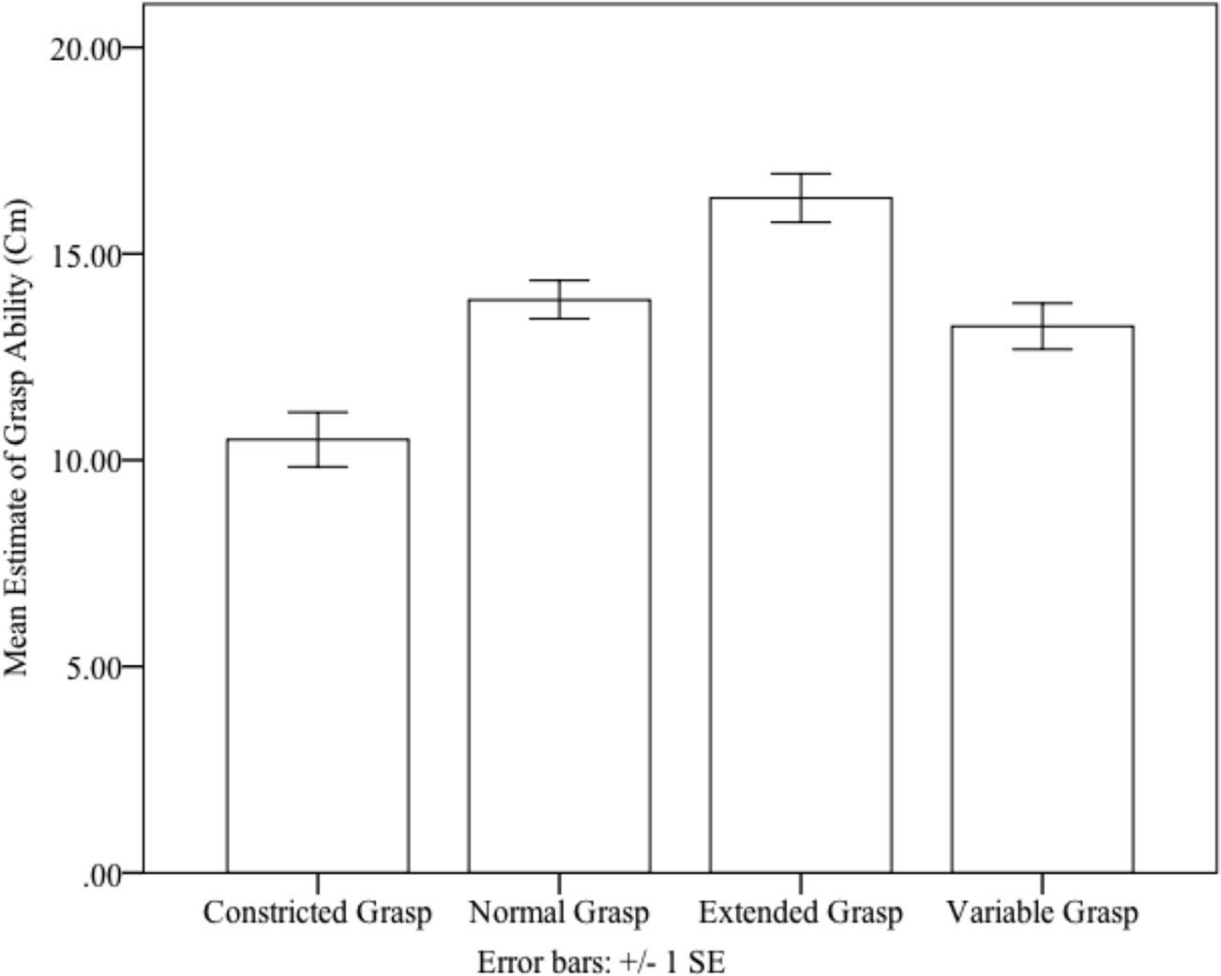
Means (and standard errors) of grasp-ability estimates for Constricted, Normal, Extended, and Variable grasp conditions. Error bars represent 1 ± 1 SEM, calculated within subjects for each condition

A Greenhouse-Geisser correction was applied to correct for violations of sphericity. Analysis revealed a significant main effect of action capability on estimate of grasp ability, *F* (1.957, 54.807) = 27.24, *p* < .001, *ŋp*^*2*^ *=*. 49. Grasping ability estimates were larger in the extended grasp condition (*M =* 16 cm*, SE* = .6 cm) than in the normal (*M* = 14 cm*, SE =* .5 cm*, p* < .001), and constricted grasp (*M* = 11 cm*, SE =* .7 cm*, p* < .001) conditions. Grasp-ability estimates in the variable grasp condition (*M* = 13 cm, *SE* = .6 cm) were larger than the constricted grasp condition (*p* = .006) and smaller than the extended grasp condition (*p* < .001). However, they were not significantly different from the normal grasp condition (*p* = .900; see Fig. 3).

A significant main effect of Hysteresis, *F* (1, 28) = 28.07 , *p* < .001, η*p*^2^ = .50, was observed. Participants estimated grasping ability to be larger when the starting block began the large (*M* = 14 cm, *SE* =.4 cm), than when the starting block began small (*M* = 13 cm, *SE* = .4 cm). No significant interaction between hand size and hysteresis was found, *F* (3, 84) = 2.07, *p* = .110.

## Experiment 2

The findings from Experiment 1 can be taken to indicate that when participants experience all action capabilities with equal probability, the perceptual system employs a mechanism based on weighted averages. If this is correct, then systematically varying experience to favour the extended grasp should shift participants perceptions to more closely reflect the extended grasp condition. Therefore, in Experiment 2 participants gained experience with the constricted and normal grasps 25% of the time and the extended grasp 50% of the time in the variable grasp condition prior to estimating their grasping ability.

## Method

### Participants

G*Power software (Faul et al., 2007) was used to perform an a priori power analysis to ascertain the required sample size to achieve adequate power. The required power (1- β) was set at .80 and the significance level (α) was set to .05. Based on Lin et al. (2020) Experiment 2, where a similar VR paradigm was used to analyse the influence of systematic variability in reaching ability, we anticipated a large effect size of 0.7. This was deduced as this study obtained a ηp^2^ of .34 with a sample of N =21. For the frequentist parameters defined, a sample size of N = 3 is required to achieve a power of .80 at an alpha of .05.

Thirty Lancaster University students (eight males) aged between 18 and 35 years (*M*_*age*_ *=* 19.72*, SD*_*age*_ = 3.16), participated. All participants received course credit for their participation. Twenty-six participants were right-handed, three participants were left-handed, and one participant was mixed-handed. The one mixed-handed participant elected to complete the study with their right hand. All participants had normal or corrected-to-normal vision and had no known medical history of motoric or rheumatic difficulties. All participants provided informed consent.

### Stimuli and apparatus

**Fig. 4:**
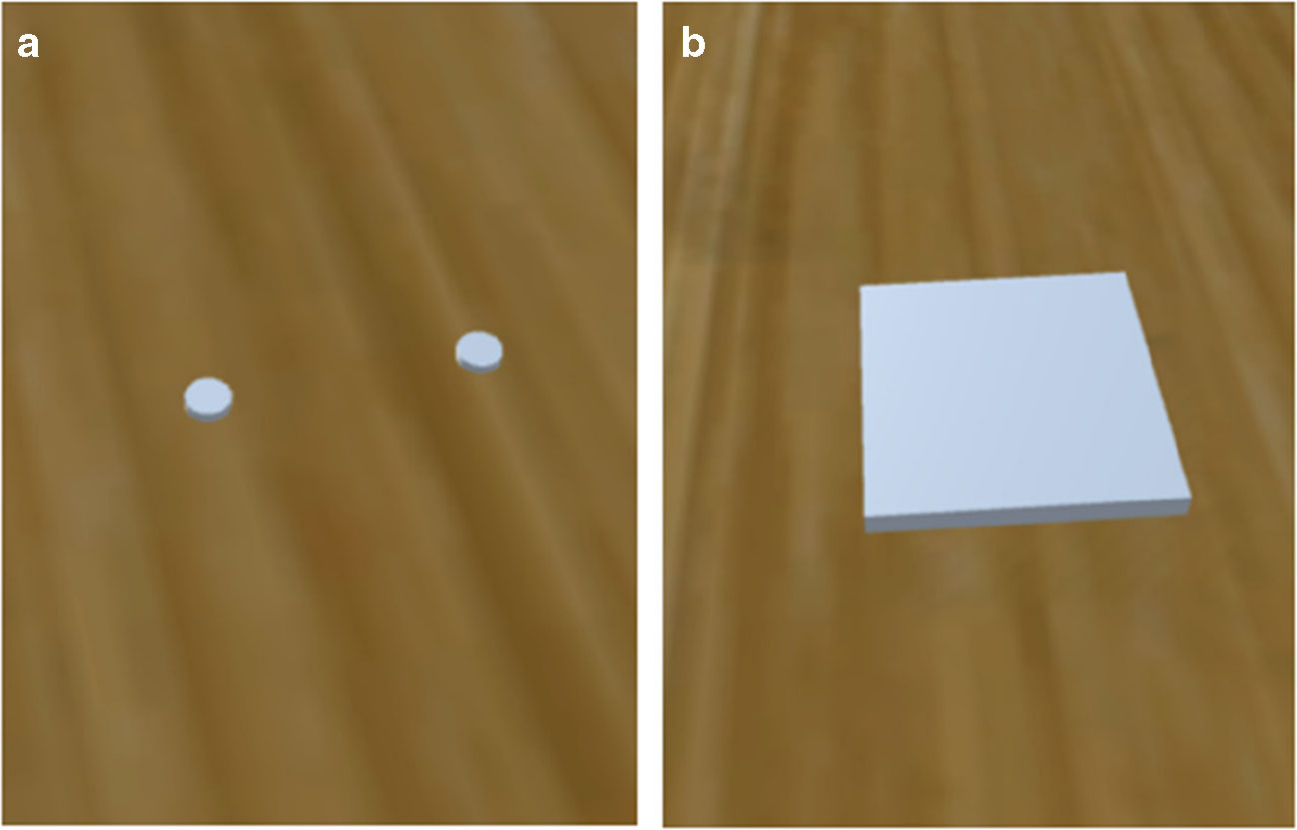
An example of the environment presented to participants in the calibration trials (**A**) and the block size manipulation trials (**B**)

The stimuli and apparatus used in Experiment 2 were consistent with those used in Experiment 1. Only minor aesthetic differences in the virtual environment (Fig. 4A) and colour of the dots in calibration trials (Fig. 4B) occurred.

### Procedure

The procedure followed in Experiment 2 was consistent with the procedure followed in Experiment 1, with the only difference being the proportion of experience participants gained with each hand size in the variable grasp condition. In Experiment 2, the participants’ experience with each of the three grasps was systematically weighted so that participants experienced the constricted hand size 25% of the time, the normal hand size 25% of the time, and the extended hand size 50% of the time.

As in Experiment 1, each condition required participants to complete the calibration and test phases, whereby the test phase in each condition included eight trials. Therefore, eight estimates of grasp ability for each experimental condition were obtained from each participant.

## Results

One participant was excluded prior to analysis as the results obtained were ±2 SD away from the mean. To analyse the influence of random variability in perceptual motor experience on perceptions of grasping ability, a 4 × 2 repeated-measures ANOVA: 4 (Grasp ability: Constricted, Normal, Extended, Variable) × 2 (Block Size: Small, Large) was conducted.

**Fig. 5:**
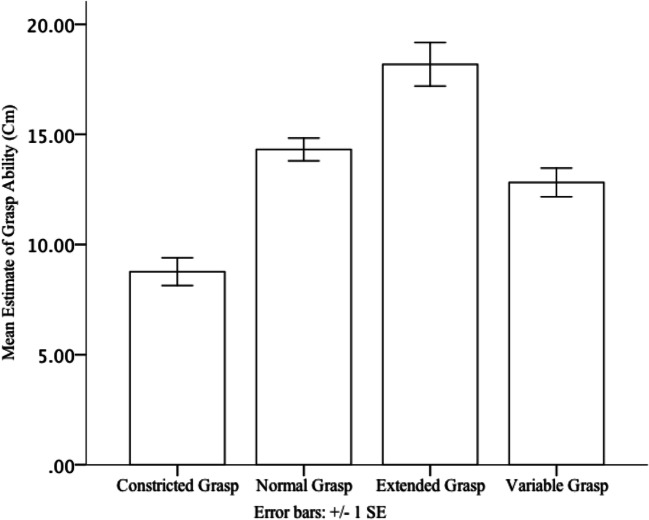
Means (and standard error) of grasp-ability estimates for Constricted, Normal, Extended, and Variable Grasp Conditions. Error bars represent 1 ± 1 SEM, calculated within subjects for each condition

A Greenhouse-Geisser correction was applied to correct for violations of sphericity. Analysis revealed a significant main effect of hand size on estimate of grasp ability, *F* (2.155, 60.33) = 34.317, *p* < .001, *ŋp*^*2*^*=*. 551. Grasp ability estimates were larger in the extended grasp condition (*M =* 18 cm*, SE* = 1 cm) than in the normal *( M* = 14 cm*, SE =* .5 cm*, p* = .002) and constricted grasp *(M* = 9 cm*, SE =* .6 cm*, p* < .001) conditions. Grasp-ability estimates in the variable grasp condiiton (*M* = 13 cm, *SE* = .7 cm) were larger than the constricted grasp condition (*p* = .001), and smaller than the extended grasp condition (*p* = .001). However, estimates of grasp ability were not significantly different from the normal grasp condition (*p* = .346; see Fig. 5).

A significant main effect of hysteresis was observed, *F* (1, 28) = 34.853, *p* <.001, *ŋp*^*2*^ *=* .555. Estimates of grasp ability were larger when the block initially started large (*M* = 14 cm, *SE* = .4 cm) than when the block initially started small (*M* = 13 cm, *SE* = .4 cm). No significant interactions were found, *F* (3, 84) = 3.682, *p* = 0.15.

## Discussion

The effect of random and systematic variability in perceptual-motor experience for grasping ability on individuals’ perceptions of their action boundaries was examined in two experiments. Consistent with existing literature, the results showed that when perceptual motor experience was altered consistently (e.g. the constricted, normal, and extended grasp conditions), participants’ perceptions of their action boundaries altered to reflect the motor experience gained.

Regarding variable perceptual motor experience for grasping, the results obtained here indicate that when variability is random, participants appear to consider all experience and calibrate to the weighted average, resulting in similar estimates to normal experience. Conversely, when variability is systematic, favoring an extended grasp, participants’ perceptions of grasp ability appeared to disregard the amount of perceptual-motor experience gained with each action boundary and calibrate to the middle action boundary. Although participants had more perceptual-motor experience with the extended-grasp, participants’ subsequent perceptions of their action boundary for grasping did not significantly differ from normal grasp condition and were significantly smaller than the extended grasp condition. Therefore, in circumstances in which one’s action capability is systematically varied to favour extended grasping capabilities, the perceptual system appears to calibrate to the average action boundary by type.

These findings may indicate that participants were unable to effectively calibrate to the variable hand condition and estimated their capability for future action inconsistently. Through this, the average of randomly selected estimates would align with the middle hand size condition. In this case, we would expect more variance in the variable condition than in the other conditions. However, the variances obtained across all conditions (*SE*_VariableCondition_ = .007 m; *SE*_ExtendedCondition_ = 1 cm; *SE*_NormalCondition_ *=* .5 cm; *SE*_Constricted_ *=* .6 cm) are largely similar. Thus, we find it unlikely that our results are due to individual differences in uncertainty.

Instead, it seems that when perceptual-motor experience is systematically varied, favouring an extended grasping capability, perceptual-motor experience is considered by type, rather than by frequency. Participants may disregard the amount of perceptual-motor experience with each action boundary and focus on variance by type, and subsequently calibrate to the average action boundary experienced by type. Here participants may disregard that they gained more experience with the extended grasp, and calibrate to the action boundary that is the average of the three types of grasp experienced, the normal grasp. This approach falls in line with a Bayesian stance, whereby information is prioritised in relation to intended actions as well as the frequency of experience (Weiss et al., 2002). Specifically, the type of perceptual-motor experience is of higher priority than the frequency of each type of perceptual-motor experience.

Alternatively, the perceptual system may employ a heuristic that allows for the selection of the middle action capability in terms of type. As in both experiments participants experienced three sets of action capabilities, 50%, 100% or 150%, a “take the middle” approach would lead participants to estimate future actions in accordance with the normal hand size. This heuristic would enable individuals to achieve some of the error minimization of applying a Bayesian inferencing approach while sacrificing accuracy for a reduction in computational cost.

Notably, the sample recruited here was restricted to young adults (Range_age_ = 18–35 years). As action capabilities develop from infancy into early adulthood and then relapse into late adulthood (Leversen et al., 2012), presumably the participants sampled here are in the most stable developmental phase of their action capabilities. Previous research has shown that adults are more accurate than children at perceiving their maximal vertical and horizontal reaching ability (Plumert, 1995) and aperture passing abilities (Franchak, 2019). Additionally, the ability to effectively use experience to recalibrate one’s perceptions of one’s action capabilities has been observed to increase as a function of age (Franchak, 2019). Furthermore, of particular relevance to the methodologies employed here, Creem-Regehr et al. (2019) observed that when placed in a virtual environment, children underestimated the width of their maximum crossable gap compared to adults. Interestingly, when participants completed the same task in the real world there was no difference between adults and children’s perceptions, thereby indicating that virtual environments may have a unique influence on individual’s perceptions. These trends support an age-modulated mechanism for determining the probability of future action. Therefore, future research utilising a wider age range to investigate any potential age-modulated effects on individuals’ perceptions of their action capabilities following variability in perceptual-motor ability is necessary.

As this series of studies considers only the effect of random and systematic variance favouring an extended grasp, it would be unreasonable to assume that the results obtained here can be generalized to the selection of one’s action boundary following all types of variability, for example, systematic variability favouring constricted grasping capabilities. Corroborating this, Lin et al. (2020) observed that individuals’ general bias towards liberal estimations of one’s action boundary following variability in reaching ability can be somewhat reduced by systematically biasing variability to favour a constricted action capability. Therefore, analyses of the influence of systematic variability, favouring a constricted grasping capability, are required.

As the results obtained here regarding grasping ability are incongruent with the results obtained regarding reaching ability (Lin et al., 2020), one may assume that the mechanism employed by the perceptual system in the face of variable perceptual motor experience may be contingent on the action in question. Specifically, we observed that when perceptual motor experience for grasping is randomly or systematically varied to favour an extended grasp, participants appear to disregard the frequency of experience and calibrate to the middle action boundary by type. Conversely, Lin et al. (2020) observed that regardless of the nature of variance, be it completely random or systematically varied to favour either a constricted or an extended grasp, individuals have a bias towards liberal estimations. As different actions have differential demands upon the body (Jeannerod, 1996) and carry with them differential cost-benefit ratios (Franchak & Adolph, 2014a), employing one blanket mechanism would not be flexible enough to accommodate a range of actions in various contexts. Rather, selection of the most appropriate action-specific mechanism to employ, considering associated risks of actions, appears more intuitive.

In summary, these studies demonstrate that manipulation of perceptual-motor feedback from virtual bodies influence one’s subsequent perceptions of one’s action boundaries. When perceptual-motor feedback is inconsistent, favoring greater experience with an extended grasping capability, the perceptual system appears to disregard the frequency of perceptual-motor experience and rather focuses on variance by type, and subsequently calibrates to the average action boundary experienced by type. Regardless of the amount of experience with different action capabilities, the perceptual system considers all possible action boundaries with equal weight when specifying the capacity for future action. However, it may be that additional factors such as age may be influencing the mechanism employed in the face of variability. Finally, differences between these results in the findings concerning reaching ability suggest that the perceptual system employs an action-specific mechanism to deal with variability in action capabilities.

## Supplementary Information

ESM 1 (SAV 6 kb)

ESM 2 (SAV 17 kb)

## Data Availability

The data and materials for all experiments are available as Online Supplementary Material. Neither Experiment 1 nor Experiment 2 was preregistered.
